# Stratifying ocean sampling globally and with depth to account for environmental variability

**DOI:** 10.1038/s41598-018-29419-1

**Published:** 2018-07-26

**Authors:** Mark John Costello, Zeenatul Basher, Roger Sayre, Sean Breyer, Dawn J. Wright

**Affiliations:** 1 Institute of Marine Science, University of Auckland, P. Bag 92019, Auckland, 1142 New Zealand; 2 United States Geological Survey, Reston, Virginia USA; 3 Esri, Redlands, California, USA

## Abstract

With increasing depth, the ocean is less sampled for physical, chemical and biological variables. Using the *Global Marine Environmental Datasets (GMED)* and *Ecological Marine Units (EMUs)*, we show that spatial variation in environmental variables decreases with depth. This is also the case over temporal scales because seasonal change, surface weather conditions, and biological activity are highest in shallow depths. A stratified sampling approach to ocean sampling is therefore proposed whereby deeper environments, both pelagic and benthic, would be sampled with relatively lower spatial and temporal resolutions. Sampling should combine measurements of physical and chemical parameters with biological species distributions, even though species identification is difficult to automate. Species distribution data are essential to infer ecosystem structure and function from environmental data. We conclude that a globally comprehensive, stratification-based ocean sampling program would be both scientifically justifiable and cost-effective.

## Introduction

Oceanographers provide information on how marine ecosystems function, including their role in the carbon cycle and climate change, and trends in the state of biodiversity. A stratified framework for monitoring biodiversity on land has been proposed based on multivariate analysis of climate variables^1^. It mapped 125 terrestrial strata, aggregated into 18 global environmental zones, primarily distinguished by temperature. However, a similar approach to sample the world ocean has not been proposed.

There are several global scale marine classifications^2,3^. However, the few that have distinguished discrete regions based on objective data analysis were limited to using satellite derived ocean colour^4,5^, plus temperature^6^ and salinity, to characterise proposed pelagic regions^7^. A statistically based classification of seabed habitats used bathymetry, slope, sediment thickness, geomorphology, surface primary production, and bottom temperature and oxygen, to identify 11 seascapes as a basis for designing a Marine Protected Area (MPA) network below 200 m depth^8^. Clustering of temperature, salinity, and oxygen for 200 to 750 m depths was combined with expert opinion to propose 33 global mesopelagic regions^9^. Regional studies have used remote sensed and *in situ* data to map regions for the Mediterranean Sea^10^ and North Pacific^11^. For the Mediterranean, a comparison of nine bioregionalisations recommended a stratified sampling within 11 regions^12^, and a three dimensional (3D) system based on the distribution of 1,100 species and seabed geomorphology in three depth zones was used to prioritise locations for a representative network of MPA^13^. Only the latter, and a bioregionalisation study of Australia^14^, included species endemicity in their classification. We propose a global, four-dimensional, stratified sampling approach, based on latitude, longitude, depth and time would be cost effective and scientifically justified. Here, we outline a 3D spatial framework based on environmental data as a first step in this process.

Danovaro *et al*.^15^ reviewed emerging government policies considering how to manage deep sea resources. They recommended the establishment of a deep-sea monitoring network that samples biodiversity and associated environmental variables. Available marine hydrographic^16^ and biological^17,18^ data decrease rapidly with depth. However, such data are needed because the deep sea is warming in the western Atlantic and in part of the Southern Ocean and cooling elsewhere in response to climate change^11^, and the fossil record shows the deep sea also responded to climate change^19,20^. The impacts of human activities have been moving to greater depths. Although fisheries production is primarily in the epipelagic zone, fishing has been going deeper for decades^21^. While the relative importance of the ocean bottom to overall ocean function is unclear, we suggest that both the sea surface and the sea bottom are key ocean boundaries with different roles in ecosystem function, which we contrast here.

The processes of gas exchange at the ocean surface, photosynthetic productivity in the underlying epipelagic zone and associated nutrient, oxygen and carbon dioxide concentrations^22^, carbon dioxide release from respiration in the deeper twilight or mesopelagic zone, and consumption of oxygen creating large low-oxygen layers in mid-depths, are of increased interest regarding measuring how much carbon is deposited into ocean sediments^23^. These analyses will need to include estimates of particle settlement, and contributions from larger materials, such as large animals, plants, and natural and artificial debris; and how benthic animals consume, bury and re-suspend such material^24^. However, this does not mean that all depths in the ocean need equal sampling effort. Here, we illustrate the spatial variation in physical and chemical variables with depth in the ocean derived from two new open access online resources, Ecological Marine Units (EMU), and the Global Marine Environmental Datasets (GMED) (see Methods for details).

## Depth gradients

**Figure 1:**
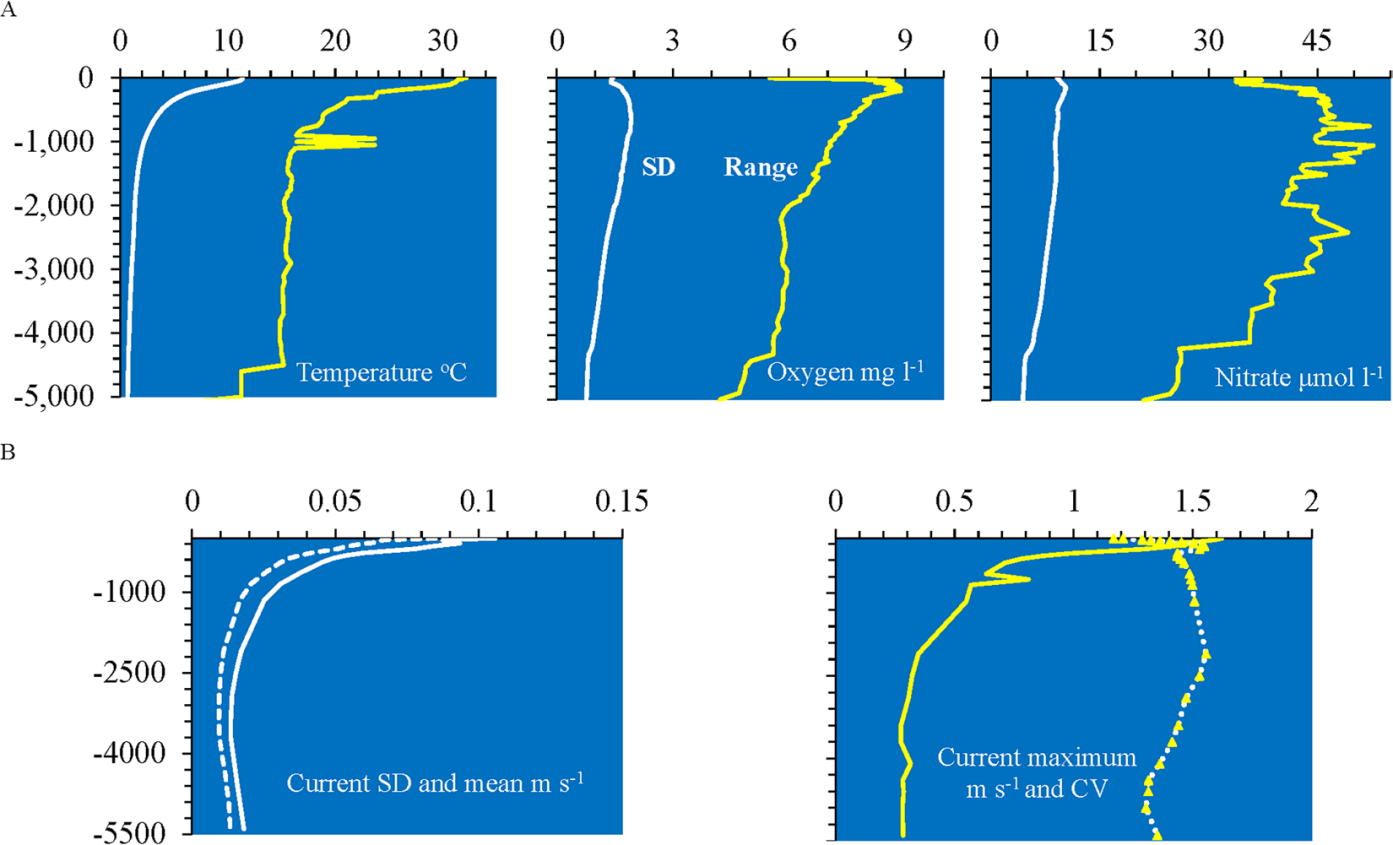
The change of environmental variables with depth. (**A**) The standard deviation (white line) and range (yellow line) of 57-year averages for temperature, nitrate, and dissolved oxygen. (**B**) Standard deviation (solid white line), average (dashed white line), maximum and range (yellow line), and coefficient of variation (dotted line and triangles) of current speed (m^s-1^), with ocean depth, summarized across all EMUs.

At least in the water column, environmental variation decreased rapidly with depth, as illustrated by temperature, oxygen and nitrate concentrations, and current velocity (Fig. 1). An exception to this generally uniform decline is noticed between 4,000 to 5,000 m (Fig. 1). This depth represents the deepest points of the Mediterranean Sea and the Gulf of Mexico which evidently are warmer and have higher oxygen and nutrient concentrations than the open oceans. At regional and local scales topographic variation and slope may influence variation in water conditions. Thus, to better capture the increased variation in environmental data from shallow waters, sampling needs to occur on a finer spatial scale at those depths and within different sea areas. This is also the case over temporal scales because seasonal change, surface weather conditions, and biological activity are highest in shallow depths.

The average, standard deviation, and maximum current speed decreased with depth (Fig. 1). Because the standard deviation was highly correlated with the mean (in contrast to the situation with other environmental variables), the coefficient of variation (CV) was calculated. The CV was *c*. 150% of the mean between 100 m to 2000 m indicating very variable velocities at these depths. The lower CV shallower than 100 m and greater than 2,500 m indicates more uniformly high and low velocities at these depths respectively.

## Surface versus seabed

**Figure 2:**
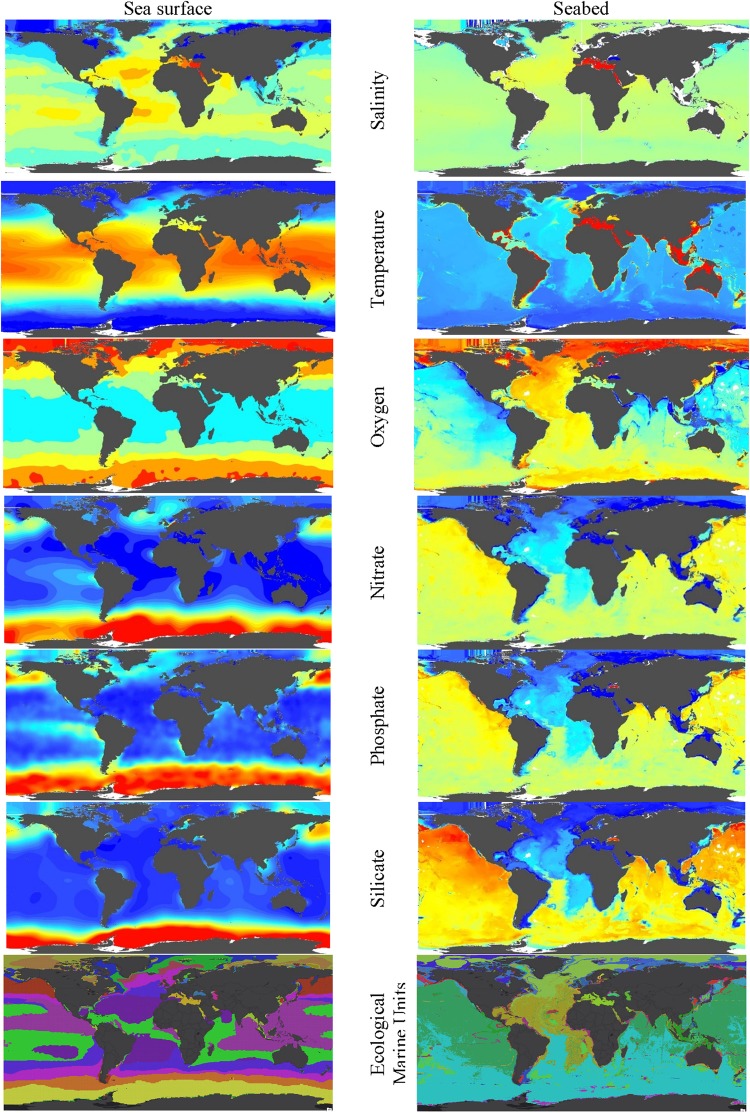
Contrast of sea surface (left) and sea bottom (right) values of environmental variables. The GMED colour scale goes from red (high) to green to blue (low), and EMU colours represent different EMU at the surface and 5,500 m depth.

**Figure 3:**
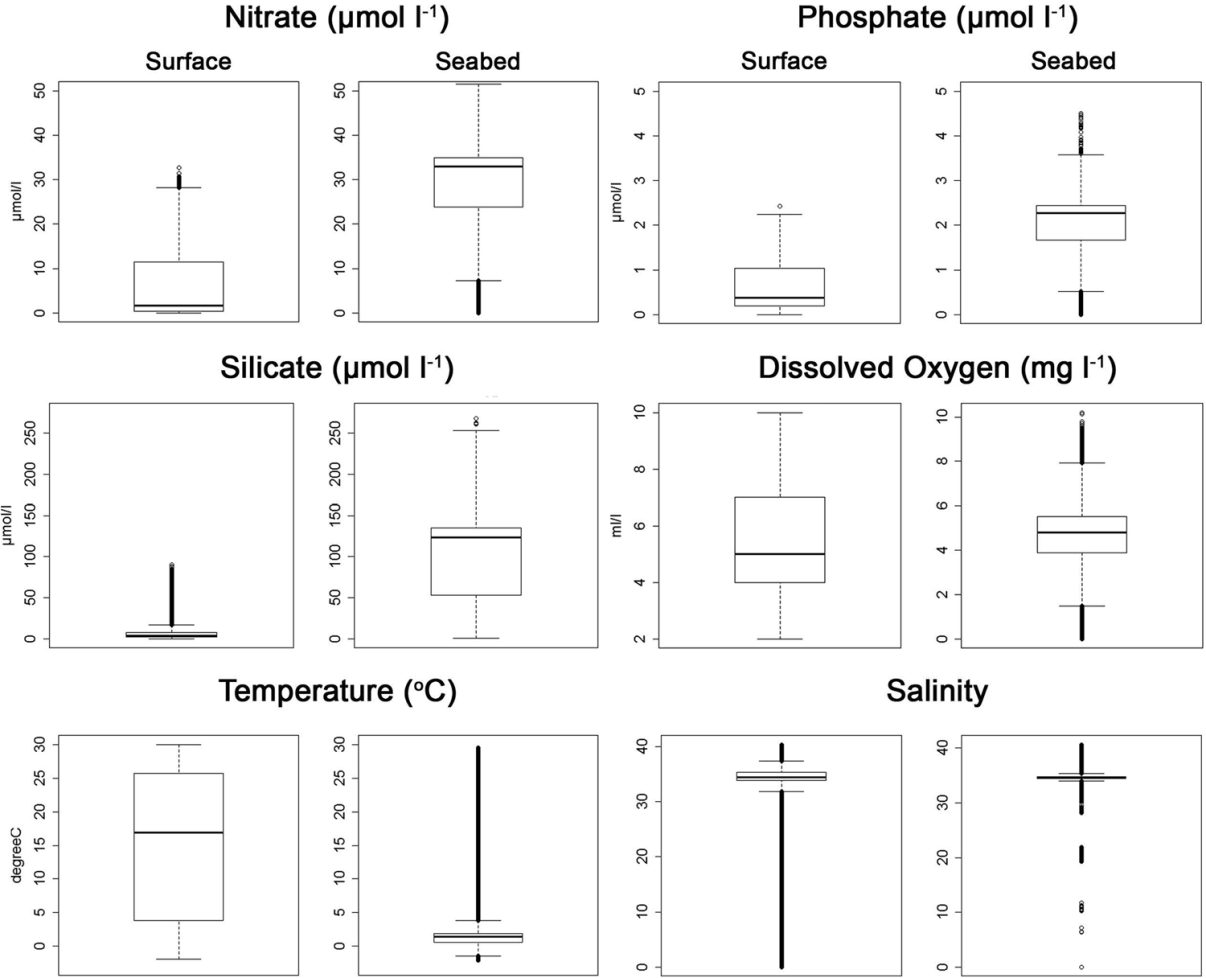
Sea surface and near sea-bed concentrations of environmental variables. Staples indicate the maximum and minimum, the box indicates the third and first quartile, the bar inside the box indicates the median and the bubbles outside the staples indicate outliers. Data from GMED.

Most variables differ strikingly between the surface and sea bottom (Fig. 2). The primary plant nutrients, nitrogen, phosphate and silicate are much higher near the seabed than sea surface, but temperature is lower, and salinity similar (Fig. 3). Median oxygen is also similar at around 5.0 to 4.8 ml l^−1^ reflecting that areas of high and low oxygen can occur in both shallow and deep waters. Moreover, the strong spatial correlations between variables within their depth zone can aid estimation of variables where field recordings may be lacking.

**Figure 4:**
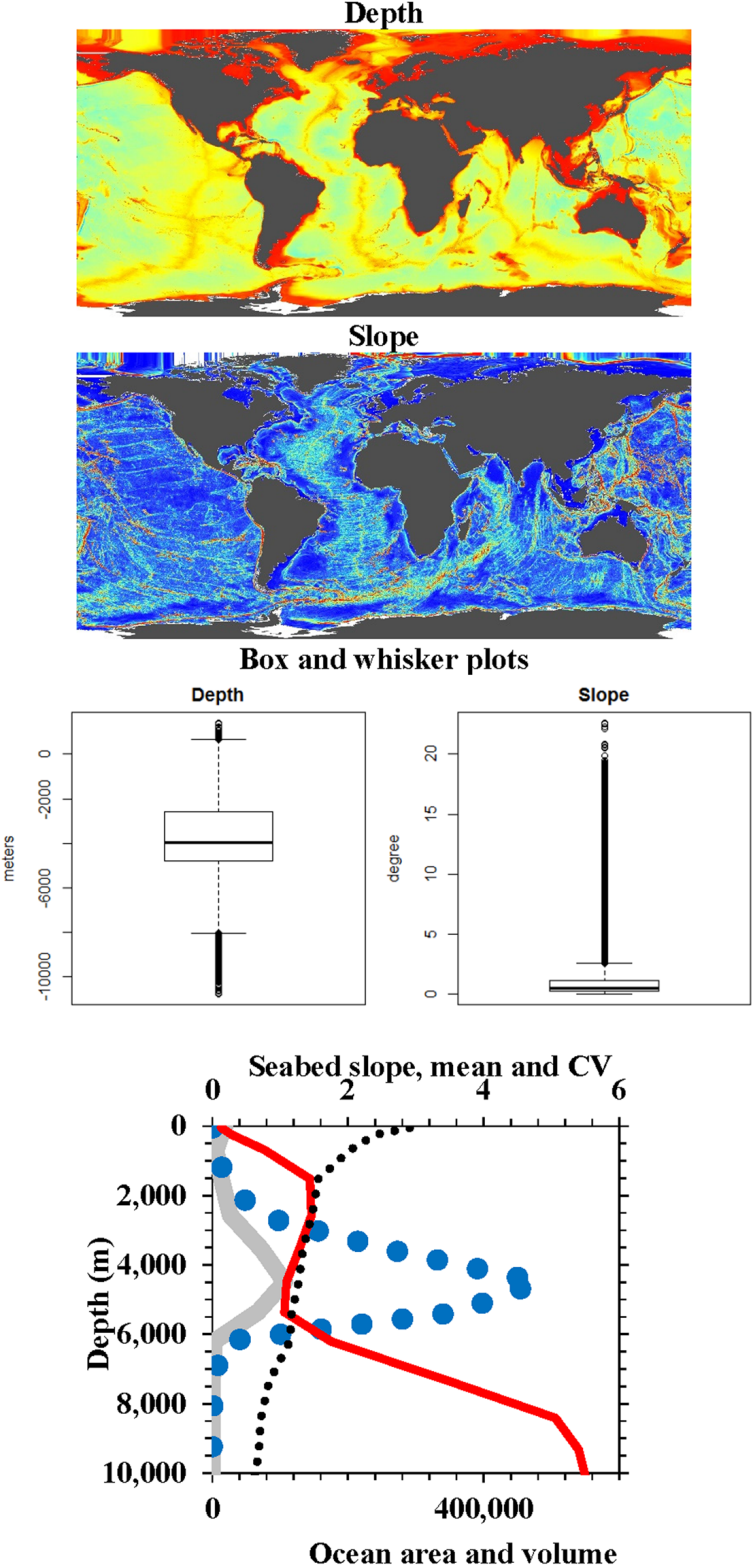
Ocean depth (m) and slope (degrees). Red is shallower and higher slope, blue is deeper and flatter slope, respectively. Because colour scales are relative values, actual median (horizontal line), 95 percentile (box), and range (vertical line) are provided as box and whisker plots (format as in Fig. 3). The graph shows how seabed slope, as both mean (narrow red line) and CV (dotted line) are low over the largest area (wide line, thousands Km^2^) and volume (large dotted line, thousands Km^3^) of the ocean between 3,000 m to 6,000 m (data from^2^). Maps from GMED.

Although the variability of water column parameters is less in the deep than shallow sea (Figs 1, 2 and 3), variation in seabed slope shows a different pattern (Fig. 4). It is highest in the deepest ocean areas but these occupy a small area and volume. Another indicator that the overall oceanic environment is more homogenous with depth is species distribution. The depth distributions and geographic ranges of marine species increase with depth reflecting environmental homogeneity, and low temperature and productivity in the deep-sea^25^. This results in a lower spatial diversity (beta diversity), species richness and endemicity in the deep-sea^26^.

## 3D framework

**Figure 5:**
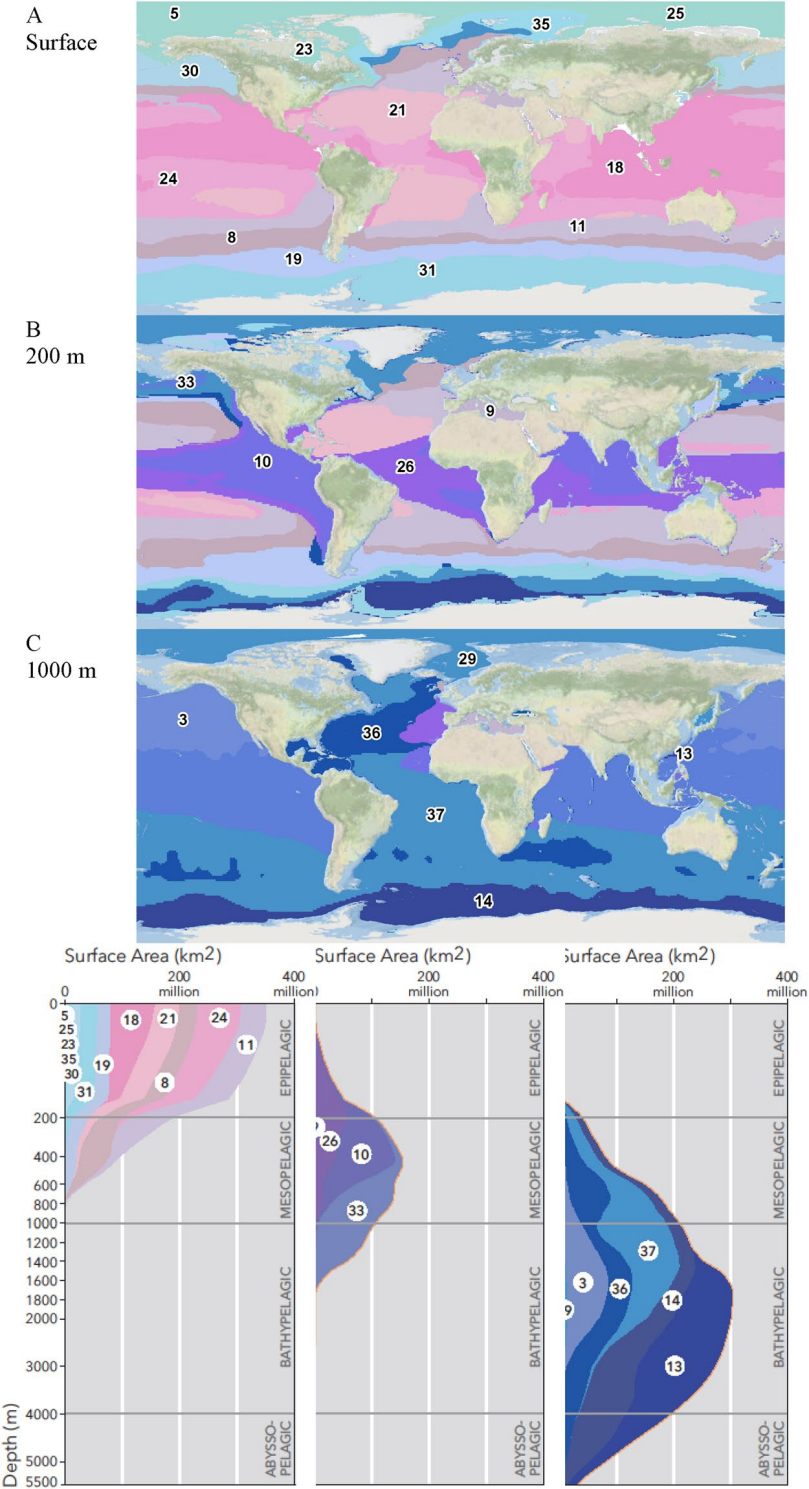
Global distribution of the 37 Ecological Marine Units. They are grouped by depth zones from (**A**) open ocean epipelagic at surface (0 m), to (**B**) mesopelagic (200 m) and (**C**) bathypelagic (1,000 m). Pink colours represent warmer, and blue colder, EMU (see^34,35^ for more details).

The spatial and depth (Fig. 5, Supplementary Material Figure S1) distribution of EMU provides a three dimensional (3D) framework to sample the oceans. The EMU analysis identified 23 primarily epipelagic (mean depth < 200 m), 7 mesopelagic (mean depth 200 to 1,000 m; EMU 1, 4, 9, 10, 26, 33, 34) and 7 bathypelagic (mean depth 1,000 to 4,000 m; EMU 3, 13, 14, 15, 29, 36, 37) units, with none primarily below 4000 m^35^. This decrease in EMU numbers with depths reflects the decreasing environmental variability in deeper waters. To get an accurate representation of the state and trends in ocean biodiversity, physical, chemical and biological data need to be collected in each EMU.

**Figure 6:**
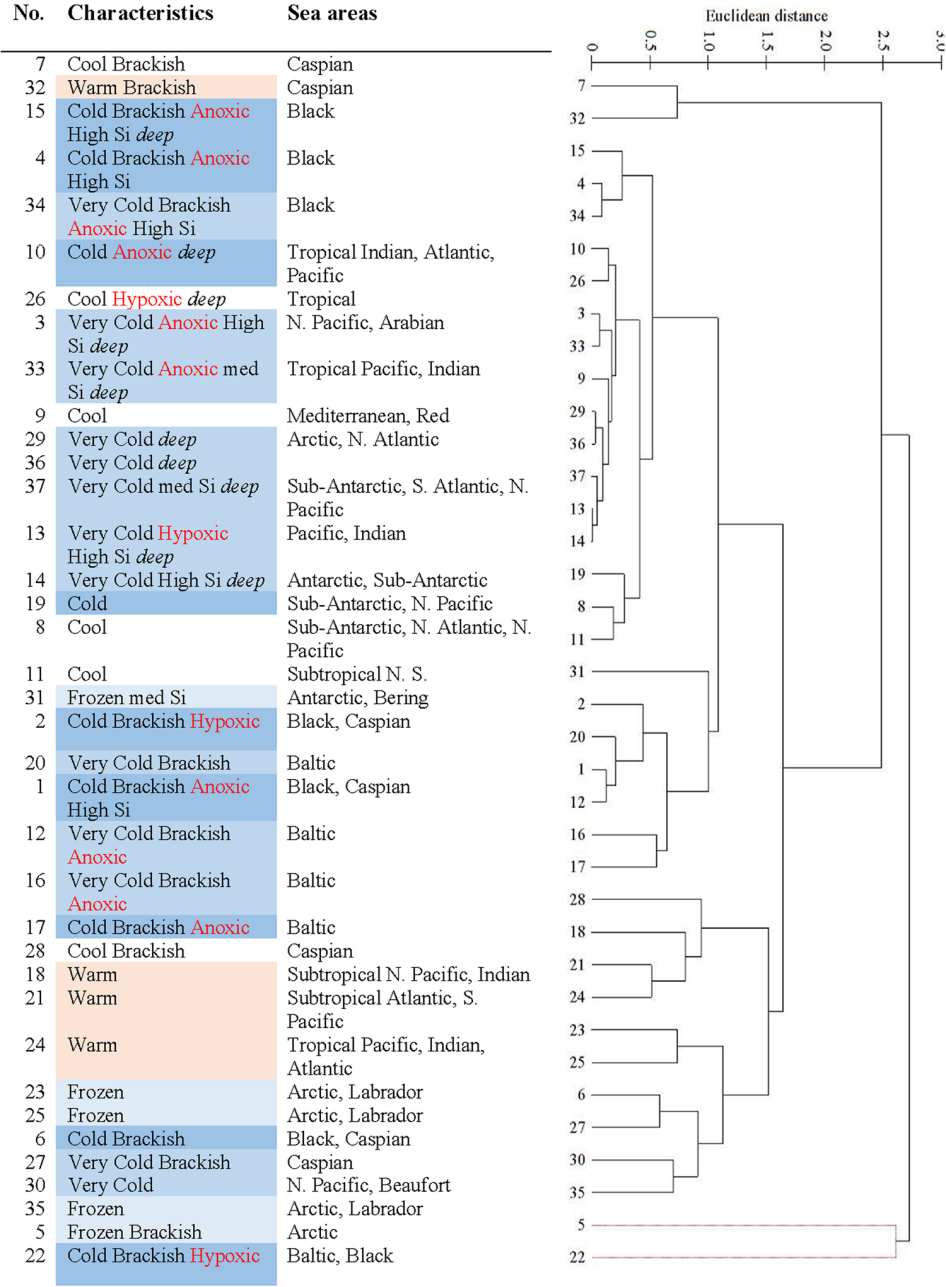
Hierachical classification of Ecological Marine Units according to the similarity of their environments based on salinity, temperature, oxygen, nitrate, phosphate, and silicate. N = north, S = south.

The EMU were clustered hierarchically based on the similarity of six environmental variables (see Methods, Fig. 6, Supplementary Material Figure S2). All EMU were found significantly different (P < 0.05, SIMPROF test in PRIMER-E^27^ except EMU 5 and 22. Cluster analysis (also using Bray-Curtis and group average on normalised data) showed the relative significance of the variables in distinguishing EMU. It found that depth was the most important and then respectively, salinity, silicate, temperature, oxygen, and equally nitrate and phosphate (Figure S2).

## Discussion

We thus propose that a spatially and temporally stratified sampling strategy will be most cost effective for exploration in the deep-sea. In accordance with decreasing environmental variability, less samples will be necessary with depth for improved characterization of the deeper water column. The EMU provide a 3D framework up to 5,500 m depth to stratify ocean sampling. However, one caveat in the use of the EMU is that their relationship to species distributions and abundance awaits more detailed analysis. It is possible that they could be aggregated, such as at a higher level on the hierarchy in Fig. 6, or need to be more finely divided, to capture spatial and temporal trends on biodiversity. For example, while EMU with a similar environment exist in different parts of the world, their species composition will vary due to geographic isolation. Contiguous EMU, such as within the Baltic, Black and Caspian Seas, may contain the same species which have adapted to the varying environmental conditions. In addition to analysis of the biological applicability of the EMU, mapping EMU with additional environmental variables and more *in situ* data will produce a more accurate framework. However, while additional data are available for the sea surface (e.g., in GMED), they are not for depth.

Although global maps of ocean geomorphology are available^28^, and there is a growing catalogue of seabed composition, such as rock, compacted sediment, and soft muds with promising methods to use these data to model wider spatial scales^29^, there remains a great deal of uncertainty about substratum composition, texture and depth on a global scale^30,27^. However, particle flux data are becoming available and considerable data can be available at national and regional scales (e.g^31,32^). Ocean sampling and observation thus needs to emphasise the surface and sea bottom environments where physical, chemical and biological variability and biodiversity are highest. To some extent, annual averages capture some of the environmental variation, but significant seabed disturbances can be episodic and extreme events may have significant long-term effects on biodiversity at all depths^33^. Thus the optimal frequency of sampling over time also merits further assessment. It is likely that this could also be stratified based on how variable environmental and biological parameters vary over time.

While satellites, acoustic methods, and other sensors are most cost-effective for collecting data, the more challenging observation of species must be emphasised^34^. Last *et al*.^14^ emphasised that species endemicity was an essential part of any classification designed to aid biological resource management. Knowing the distribution and dynamics of species is fundamental to sustainable use of biodiversity, including fisheries and fish food, and also because of the biological effects on the carbon cycle and other bio-chemical processes. Furthermore, the distribution of species signals longer-term environmental conditions. Efforts to associate species distribution data with distinct abiotic environments like EMUs will further illuminate the relationship between environmental drivers and species distributions. A global classification of marine biogeographic realms based on species endemicity is now available^28^. As expected from our knowledge of the environmental variation and productivity, it found lower species’ endemicity and thus fewer realms in open ocean and deep-sea environments than coastal. This could be cross-matched with EMU^35,36^ seascapes^8^, geomorphological units^30^, and other environmental regions to provide a fully integrated system for marine management and monitoring.

Ocean exploration benefits from international collaboration and publication of data^37,38^, as well as data products such as EMUs and GMED. The marine community has a good track record in collaboration and data management^39^, having established the Argo floats programme, a complete inventory of all marine species (World Register of Marine Species^40^), an open access database on marine species distributions (Ocean Biogeographic Information System), and partnering in oceanography^41^. The Group on Earth Observations (GEO), supported by over 100 countries, provides a world brokerage for collaboration in the ocean sciences^42^ and has initiated a Marine Biodiversity Observation Network (MBON)^43^. This synergy of field efforts will improve the quality and cost efficiency of data collection and management through mutual exchange of know-how and resources. The benefits will include a new understanding of ocean ecosystems that will inform sustainable resource use and government policies.

## Methods

The ‘Ecological Marine Units’ (EMU) provided the first three-dimensional (3D) partitioning of the ocean based on environmental variables^37,38^. The EMU represent 37 physically and chemically distinct volumetric regions in the ocean that were objectively derived from a non-supervised clustering of ocean environmental data. The variables clustered were from NOAA’s World Ocean Atlas (WoA); namely, 57 year averages of temperature, salinity, oxygen concentration, oxidised nitrogen (~nitrate), phosphate, and silicate^44–47^. Prior to analysis using Euclidean distance and group average clustering, the data were normalised by the mean value for each variable being subtracted and divided by their standard deviation so each variable had equal weight. Their lower extent is 5,500 m due to data availability. A depth of 5,500 m is sufficient to include much of the global seabed because the average depth of the ocean is approximately 3,400 m although it extends to 11,000 m^39^. This illustrates that a priority for ocean science is to provide not only more variables in 3D, but to extend them to the seabed everywhere. Using spatial data interpolation techniques, the EMU have also been attributed with current velocity data from a global model simulation hindcast representing the climatological mean for the period 2000–2012^48,49^.

The Global Marine Environment Datasets (GMED) resource is an open-access online compendium of most global scale marine data in a standardised spatial resolution^50^, and includes variables representing both sea surface and near seabed conditions. As the source for near seabed data is also WoA this is limited to about 5,500 m depth. As with the EMUs, GMED seeks to make already existing data more accessible to non-specialists, such as educators, biologists and ecologists.

### Data availability

The Ecological Marine Units are available at http://www.esri.com/ecological-marine-units and can be explored at https://livingatlas.arcgis.com/emu/ and https://www.arcgis.com/home/item.html?id = 58526e3af88b46a3a1d1eb1738230ee3. The Global Marine Environmental Datasets are available at http://gmed.auckland.ac.nz.

## Electronic supplementary material

Supplementary Information
