# Supplementary material for: Stratifying ocean sampling globally and with depth to account for environmental variability
Source: Sci Rep. 2018 Jul 26;8:11259. doi: 10.1038/s41598-018-29419-1 (PMC6062513; doi:10.1038/s41598-018-29419-1)
Supplement: Supplementary file 1 — Supplementary Information [file 41598_2018_29419_MOESM1_ESM.doc]

Stratifying ocean sampling globally and with depth to account for environmental variability

Mark John Costello, Zeenatul Basher, Roger Sayre, Sean Breyer, Dawn Wright

**Supplementary Material**


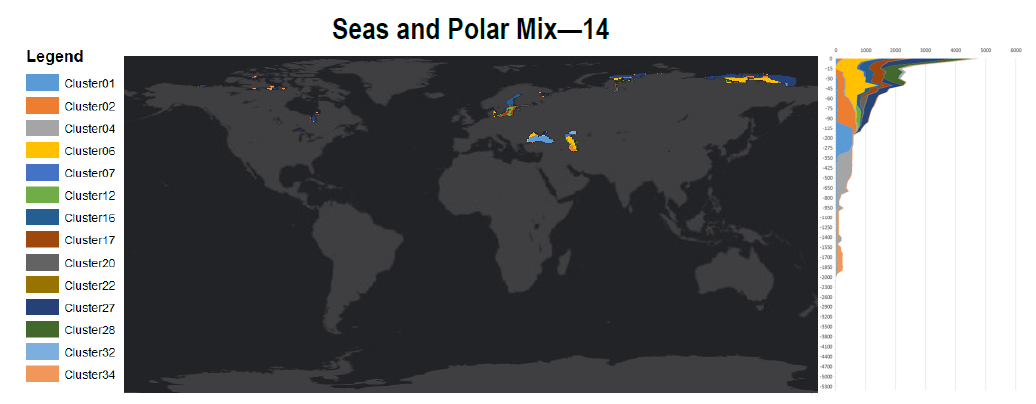


Figure S1. The distribution of the 14 coastal Ecological Marine Units (EMU) in the Arctic, Baltic, Black and Caspian Seas.


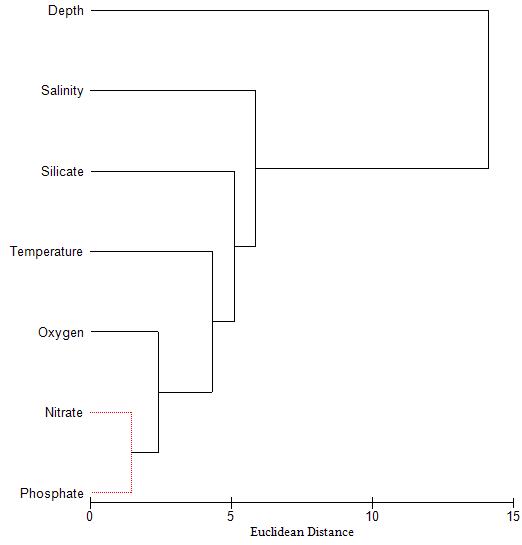


Figure S2. The relative influence of the environmental variables in discriminating between the EMU. The red line indicates no significant difference between nitrate and phosphate.
